# Association between WeChat Use and Memory Performance among Older Adults in China: The Mediating Role of Depression

**DOI:** 10.3390/bs12090323

**Published:** 2022-09-06

**Authors:** Zhiya Hua, Fangling Wang

**Affiliations:** 1 School of Government, Shanghai University of Political Science and Law, Shanghai 201701, China; 2 Shanghai Yao Lian Sheng Middle School, Shanghai 200051, China

**Keywords:** social media, depression, memory decline, memory performance, older adults, WeChat, China

## Abstract

Changes to memory performance in the course of aging may be influenced by behavioral factors. The use of social media among elderly people is increasing, but studying its effect on cognitive functions such as memory remains at an early stage of development. Meanwhile, the linking mechanisms underlying the association between social media use and memory performance, if any exist, have not been revealed. This study attempted to examine the association between the use of WeChat, the most popular social media platform in China, and memory performance among older people, and to test the possible mediating role of depression underlying this association. Data were drawn from the five-wave survey of the China Family Panel Study (CFPS), and 4929 respondents aged 60 or older (mean age = 68.19, SD = 5.84, 48.2% females) were included. Based on the descriptive statistics, the chi-squared test, Student’s *t*-test, correlation analysis, and mediation analysis were conducted. The results indicated that the usage rate of WeChat among the sample was 20.1%. After controlling for demographic variables, the use of WeChat was related to higher levels of memory performance and lower levels of depression. Moreover, depression partially mediated the relationship between WeChat use and memory performance. To maintain memory performance and promote cognitive health in the course of aging, using social media and alleviating depression merit special attention.

## 1. Introduction

Forgetfulness and memory decline are among the foremost challenges facing aging populations [1]. Memory decline involves changes in various types of memory, such as working memory and long-term memory [2,3]. Memory performance, as a holistic measure, can reflect the final outcome of various memory changes [4]. As a critical underpinning component of cognitive ability, decline in memory always contributes to failures in daily functions [5] and impaired abilities to retain new information [6], which seriously threaten the quality of life and even result in the loss of independence of the elderly [7,8]. In addition, old people’s perception and appraisal of their memory performance may cause anxiety about memory abilities and exert a negative impact on their self-concept and general well-being [9,10]. Given that most people in the world are living longer [11], it is of increasing importance to identify the contributing factors to any underlying mechanisms of memory changes in the course of aging and to explore effective strategies to maintain memory performance, or at least to delay memory decline in older people.

Changes to memory in the course of aging are the result of complex interactions between organic, neural, psychological, behavioral, and demographic factors [12]. Accordingly, researchers and clinicians have explored various techniques and approaches to attempt to delay memory decline, among which behavioral changes stand out as they can be implemented relatively easily in daily life [13]. In recent years, as more and more elderly people have begun to use social media, the association between social media use and memory performance has become an emerging research focus.

Social media refers to Internet-based communication platforms that enable users to connect and interact with other users in various ways [14]. Due to the advent of Internet technologies, using social media has become an integral part of daily digital activities. It is estimated that more than 4.26 billion people in the world used social media in 2021, and average social media usage time is 144 min per day [15]. During the last decade, the increase in older users has been a striking aspect of social media markets [16]. According to the Pew Research Center, about 45% of American adults aged 65 and older reported that they had used social media [17] (p. 5). In China, older Internet users (aged 60 and older) totaled 119 million in 2021, and 90.6% of them used social media [18] (p. 34).

Although some studies have reported that social media use may impede memory performance [19,20,21], a growing body of research has suggested that the use of social media can yield a variety of beneficial effects, such as convenient access to information [22], enhanced connectiveness and social engagement [16,23], increased perceived social support [24], diminished loneliness [25], and stimulating cognitive activities such as reading, thinking, discussing, and learning [26], all of which have been proven to be protective resources for memory [27,28]. Indeed, an abundant literature has reported that social media usage is positively correlated with memory performance [29,30,31]. For example, through a comparison between users and non-users of social media among a group of senior citizens, Kim and Kim found that the cognitive function levels of social media users, including memory capacity, were significantly higher than those of non-users [30]. Similarly, a randomized trial study of 41 older adults conducted by Myhre et al. revealed that, in comparison to the controls, those who received 8-week Facebook training sessions obtained a significant improvement in memory performance [31]. Furthermore, some studies have also explored the neurological basis of the relationship between social media use and memory performance. For instance, Kanai et al. conducted four experimental studies, finding that grey matter density in the entorhinal cortex, the area of the brain implicated in associative memory, is significantly positively related to online social network size [29]. In addition, a number of studies have revealed that certain inner characteristics of social media, such as those features that relate to their content, format, and engagement style, may help to promote memory performance [32,33,34,35]. For example, a diary study of posting behaviors on social media indicated that sharing personal experiences online facilitated memory retention [34]. Although the positive association between social media usage and memory performance has been reported, the potential processes and mechanisms linking them remain unclear. Hence, this study attempted to explore the potential mechanisms linking social media usage to memory performance.

In the process of exploring the contributing factors of memory decline, depression has recently attracted increased attention. Depression is a common emotional disorder, referring to a medical condition in which individuals experience persistent sad or empty thoughts; furthermore, it often has physical symptoms such as insomnia [36]. Substantial literature has found that memory performance is decreased among people with depression [37,38,39,40,41]. For example, in a meta-analysis of 69 studies, McDermott and Ebmeier found that the severity of depression was significantly negatively related to episodic memory [32]. Similarly, some researchers have observed that individuals with depression lack specificity in their autobiographical memories [42], which also affects their memory performance. In contrast, through the analysis of longitudinal assessments, Douglas and Porter found that relief of depressive symptoms was significantly associated with improvements in verbal memory [43]. Moreover, although conflicting results exist [44,45], some studies found that social media usage was negatively associated with depression [46,47]. For example, O’Keeffe and Clarke-Pearson reported that social media use may improve children’s psychological health, as they enable users to reduce the risk of depression through strengthening interaction with others [48]. Similarly, Grieve and colleagues found that levels of connectiveness on Facebook and severity of depressive symptoms were negatively correlated [49]. Therefore, research has linked the use of social media to a lower risk of depression, and meanwhile, a lower risk of depression has been found to be related to higher levels of memory performance. In addition, some studies reported that certain emotional variables might mediate the relationship between social media usage and memory performance [50,51]. For example, a daily dairy study conducted by Sharifian and Zahodne found that negative affect served as a significant mediator between social media use and memory performance [51]. Based on the aforementioned correlations among social media usage, depression, and memory performance, we infer that depression might mediate the association between social media use and memory performance. However, this mediating relationship has not been rigorously tested by empirical research.

In summary, changes to memory are one of the fundamental aspects of aging. As the use of social media among the elderly population has increased, the relationship between social media use and memory performance in older people has become a central topic of investigation. Some studies have found that social media usage and memory performance were positively correlated, but opposing findings have also been found. At the same time, the linking mechanisms underlying this potential association remain unclear. Therefore, more studies are needed to further understand the association between social media use and memory performance. In addition, although a growing number of older Chinese adults have begun to use social media [18] (p. 34), little research has empirically studied the association between social media usage and memory performance among them. The objective of the present study was to examine the association between social media usage and memory performance and to test for the mediating role of depression through focusing on WeChat use among older Chinese adults. WeChat is the most popular social media platform in China. It was first released by Tencent Holdings Limited in 2011 and was initially a messaging app. Although some extended applications such as online payment, financial management, food-delivery, and e-commerce were gradually integrated into it, the core functions of WeChat are still communicating with others and seeking and sharing information through reading or posting messages. Messages can be transmitted on WeChat using various formats, such as texts, voice recordings, photos, and videos. Hence, it is seen as a kind of social media similar to WhatsApp and Facebook [52,53]. WeChat has grown into the most influential social media giant in mainland China. According to the 49th Statistical Report on China’s Internet Development issued in February 2022, WeChat boasted over 450 million daily active users in 2021 [18] (p. 45). Similarly, a survey conducted by the Chinese Academy of Social Science in 2021 found that WeChat was the most commonly used social media among elderly people in China [54].

**Figure 1:**
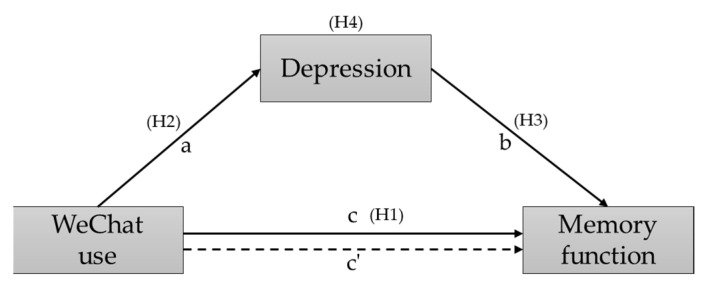
The hypothesized model concerning the relationships between WeChat use, depression, and memory performance.

Specifically, through an investigation of a sample of older adults in China, the following four hypotheses were tested: (1) WeChat use and memory performance are positively associated; (2) WeChat use is negatively related to depression; (3) Memory performance is negatively associated with depression; (4) Depression mediates the relationship between WeChat use and memory performance. The hypothesized model is shown in Figure 1.

## 2. Materials and Methods

### 2.1. Data Source and Research Sample

Data used in the present study were drawn from the five-wave survey of the China Family Panel Study (CFPS), which was a longitudinal survey of a nationally representative sample in China. The main objective of CFPS was to collect data regarding various aspects of China’s society, such as the economy, demography, education, and health at the levels of the individual, family, and community, and to provide an empirical basis for contemporary Chinese studies and public policy analyses [55]. The CFPS was conducted by the Institute of Social Science Survey (ISSS) at Peking University in collaboration with the Survey Research Center at the University of Michigan. More information concerning the study design and sampling procedures can be found elsewhere [56,57]. The baseline survey, covering about 16,000 families in 25 provincial regions across China, was implemented in 2010. According to the protocol, the tracking survey was implemented every two years from 2012 onwards, and all members of the baseline-surveyed families and their descendants became the respondents. The five-wave survey was completed in 2020, and its data at the individual level were released in December 2021. The CFPS data are openly accessible on the website of the ISSS at Peking University (http://www.isss.pku.edu.cn/cfps/ (accessed on 22 May 2022)). Our institutional review board determined that this study did not need a full board review as the CFPS project had been reviewed and approved by the Biomedical Ethics Committee of Peking University (IRB00001052-14010) [58].

The sample analyzed in this study was derived through the following steps. First, 6976 people aged 60 or older among the total 28,590 respondents were obtained from the five-wave CFPS individual level survey dataset. Second, of these, the 1881 people who did not provide an answer for either WeChat use or memory performance were dropped. Third, 166 people with depression scores beyond the scale range were excluded. The anonymous data of the remaining 4929 older adults constituted our final analysis figures.

### 2.2. Variables and Measures

#### 2.2.1. Demographic Variables

Some demographic variables, such as age, gender, educational attainment, marital status, and household registration type were analyzed as covariates.

#### 2.2.2. WeChat Use

The use of WeChat was assessed by the survey item: “Do you use WeChat? (1 = yes, 0 = no)”. The respondents who answered “yes” to this question were defined as WeChat users in this study. Accordingly, those who answered “no” were defined as non-users of WeChat.

#### 2.2.3. Depression

Depression was evaluated by an eight-item scale adapted from the Center for Epidemiological Studies Depression scale (CES-D) [59]. The items were: “I am in a low spirit”, “I find it difficult to do anything”, “I cannot sleep well”, “I feel happy”, “I feel lonely”, “I have a happy life”, “I feel sad”, and “I feel that I cannot continue with my life”. Responses to each item were arranged on a four-point Likert rating scale from 1, “never (less than one day in the past week)” to 4, “most of the time (5–7 days in the past week)”. The scores of the two negative items (4 and 6) were reversed, and then the scores for all 8 items were added to obtain a total score, which ranged from 8 to 32, with a higher total score indicating a higher level of depressive symptoms. The CES-D has shown good reliability and validity among Chinese people [60,61]. Cronbach’s alpha of the 8-item scale was 0.778, suggesting an acceptable internal consistency in the current sample.

#### 2.2.4. Memory Performance

Memory performance was assessed by the survey question: “Are you able to remember the important things that happen to you within a week?”. The answers were arranged on a five-point Likert scale (1 = Remember only a little bit, 2 = Remember a few of them, 3 = Remember half of them, 4 = Remember most of them, 5 = Remember all of them). Because the answers to the question included five choices, according to researchers’ recommendations [62,63], memory performance was treated as a continuous variable in this study. The higher the score, the higher the level of memory performance reported by the respondents.

### 2.3. Statistical Strategies

First, descriptive statistics was conducted to depict the sample and main characteristics of the variables of interest. Meanwhile, the chi-squared test was performed to determine whether differences in WeChat usage rate existed among older adults with different demographic characteristics. Second, Student’s *t*-test was performed to examine the differences in depressive symptoms and memory performance between WeChat users and non-users. Third, a bivariate correlation analysis was performed to examine the associations among WeChat use, depression, and memory performance among the respondents. Specifically, point-biserial (for dichotomy-continuous variables) and Pearson’s (for two continuous variables) correlations were adopted, respectively, according to the types of variables. Finally, to test and verify the mediation effect of depression between WeChat use and memory performance, the causal step regression method [64] and the bootstrapping method proposed by Preacher and Hayes [65,66] were conducted. The bootstrapping estimation was executed using the PROCESS macro for SPSS (version 3.5), designed by Hayes [67]. According to Hayes’ recommendations [67], PROCESS model 4 (the simple mediation model) with 5000 resamples was utilized, and the mediation effect was determined to be significant if the 95% confidence intervals of indirect effect did not contain zero. To control type Ⅰ and type Ⅱ errors, the significance level was set as *p* < 0.05. All analyses were performed by SPSS 26.0 (SPSS Inc., IBM, Chicago, IL, USA).

## 3. Results

### 3.1. Description of the Sample

The mean age of the 4929 respondents was 68.19 ± 5.84 years old. A total of 95.2% of them belonged to the 60–79 age group, while 4.8% of them were over 80 years old. In the sample, the percentage of males was slightly higher than females (51.8% vs. 48.2%). Most of the respondents (82.8%) were married and had a spouse, and 14.5% of them were widowed. With respect to educational attainment, 40.3% of them were illiterate or semi-illiterate, and those who received primary school, junior high school, and senior high school education accounted for 22.5%, 21.4%, and 12.9%, respectively. Only 2.8% of them completed higher education. Additionally, nearly two-thirds of the respondents (67.8%) were registered as agricultural residents (Table 1).

### 3.2. The Usage Rate of WeChat

The overall WeChat usage rate among the current sample was 20.1% (993/4929). The results of chi-squared tests demonstrated that there were significant differences in WeChat usage among older adults with different demographic characteristics (Table 1). Specifically, males used WeChat more than females among the sampled older adults. The WeChat usage rate among older adults aged between 60 and 79 was higher than that among the oldest adults. Moreover, those who were divorced used WeChat more than other older adults. In addition, the WeChat usage rate increased with increases in education level.

### 3.3. Comparison between Users and Non-Users of WeChat

As reported in Table 2, the mean depression scores of WeChat users and non-users were 12.44 ± 3.96 and 13.89 ± 4.61, respectively. The result of Student’s *t*-test (t = −9.138, *p* < 0.001) indicated that the difference in depression between users and non-users of WeChat was statistically significant, showing that the older adults who used WeChat had lower levels of depressive symptoms than those who did not use WeChat.

Additionally, the mean memory performance scores of WeChat users and non-users were 3.10 ± 1.27 and 2.45 ± 1.32, respectively. Similarly, Student’s *t*-test (t = 14.075, *p* < 0.001) demonstrated that the difference was statistically significant, namely, WeChat users among older adults had higher levels of memory performance than the non-users.

### 3.4. Correlations between the Variables of Interest

The ranges, means, standard deviations, and correlation coefficients between the variables of interest are reported in Table 3. The results show that WeChat use and memory performance were significantly positively correlated (r = 0.197, *p* < 0.01). Moreover, depression was significantly negatively associated with both WeChat use (r = −0.129, *p* < 0.01) and memory performance (r = −0.238, *p* < 0.01).

### 3.5. Mediation Analysis

To test the possible mediation effect of depression between WeChat use and memory performance, the classic method of causal step regression was adopted. To control the influences of demographic variables, gender, age, marital status, educational attainment, and household registration type were taken into consideration as covariates. First, the standardized coefficient from WeChat use to memory performance was calculated, and the result (β = 0.279, *p* < 0.001) demonstrated that the total effect (path c) was significant. Second, the results of regression analysis showed that the effect of WeChat use on depression (β = −0.184, *p* < 0.001) and the effect of depression on memory performance (β = −0.190, *p* < 0.001) were significant. Third, when the hypothesized mediator (depression) was added to the model, the direct effect of WeChat use on memory performance (path c’) was also significant, but its value decreased from 0.279 to 0.244 (Table 4). All these results indicated that, after controlling for demographic variables, depression exerted a partial mediation effect on the relationship between WeChat use and memory performance.

To verify the mediation effect of depression, the bootstrapping method was adopted. Model 4 of the PROCESS macro with 5000 resamples was utilized, and gender, age, marital status, educational attainment, and household registration type were treated as covariates. The results (Table 5) indicated that the total, direct, and indirect effects of the hypothesized model were significant. Therefore, the mediation effect of depression between WeChat use and memory performance was verified.

## 4. Discussion

The present study examined the association between WeChat use and memory performance among older adults, and it tested the possible mediating role of depression between WeChat use and memory performance through the analysis of a nationally representative sample in China. The analysis results demonstrated that the usage rate of WeChat in the sample was 20.1%, considerably lower than the social media usage rate of elderly people in the U.S. (45%) [17] (p. 5) and the global average level (59.0%) [68]. It is important to note that only the most popular social media platform in China (WeChat) was considered in the current study, while the actual social media usage rate in older Chinese adults may be higher. Meanwhile, the usage rate of WeChat varied considerably according to the demographic characteristics of the elderly population, such as gender, age, marital status, educational attainment, and household registration type.

Moreover, through bivariate correlation analysis, we found that WeChat use positively correlated with memory performance, suggesting that the older adults who used WeChat had higher levels of memory performance. The comparison between WeChat users and non-users via Student’s *t*-test verified this finding: the mean memory performance scores of WeChat users among older adults were significantly higher than those of the non-users. This finding suggests that social media users remember more past events than non-users, and it extends previous studies suggesting that social media usage exerts beneficial effects on the cognitive functions of older adults [29,30,31]. However, it should be noted that due to the opposing findings produced by existing studies, the results of the current study also oppose the findings of previous studies [19,21]. For example, through both naturalistic and controlled studies, Tamir and colleagues found that individuals who recorded and shared their experiences using social media displayed poorer memory performance than those who did not use social media [21]. Some researchers have argued that the inconsistency of these findings may stem from the usage styles of different age groups [69]. Social media use usually involves multi-tasking; the elderly tend to utilize their basic functions such as communication and interaction with others, whereas the younger population likes to treat them as convenient and comprehensive Internet platforms to seek and share information. Studies have suggested that excessive access to information via social media serves as a substitution effect on memory [70]. Additionally, over-reliance on social media to handle various activities may cause anatomical changes in parts of the cerebral cortex that relate to memory [21]. Therefore, to clarify the exact relationship between social media use and memory performance, deeper investigation regarding the use patterns among various social groups is needed in the future.

In addition, in this study, the correlation analysis indicated that WeChat use was negatively correlated with depression. The older adults who used WeChat were less likely to suffer from depressive symptoms. In the same line, Student’s *t*-test verified this finding. The mean depression scores of WeChat users among the current sample were significantly lower than those of the non-users. This finding is consistent with previous studies [46,47,49]. For example, Qu et al. analyzed a sample drawn from a cross-sectional survey of middle-aged and older people in China and found that a significantly lower incidence of depression was associated with social media usage [71]. Nonetheless, conflicting findings also exist in this field [44,45]. For example, a study of 1787 young American adults aged between 19 and 32 found that depression was positively associated with the time spent on social media [44]. Conflicting findings related to depression might be partially attributed to various social media engagement styles and usage frequency [72,73], which highlights the need to further examine the relationship between depression and social media usage style and frequency. Meanwhile, through correlation analysis, we found that depression was negatively related to memory performance, which also supports previously reported findings [74,75]. For example, a study conducted by Zahodne and colleagues among 2425 initially non-demented older adults found that depression was a significant predictor of memory decline [75].

Furthermore, our analysis indicated that depression exerted a partial mediation effect on the relationship between WeChat use and memory performance. This finding confirmed our hypothesis: namely, that the older adults who used WeChat were at lower risk of depression, which, in turn, was beneficial in increasing their memory performance. This finding was consistent with previous studies [50,76]. For example, through an investigation of 3036 senior high school students in China, Sha and Dong analyzed the association between TikTok use and memory loss, and they found that depression partially mediated the relationship [50]. Through investigating older adults in China, this study adds new empirical evidence to the mediating effect of depression between social media use and memory performance. One possible explanation of the mediation effect of depression can be provided by the model of corticosteroids [77]. According to this theory, the level of corticosteroids rises during a depressive episode, and the hippocampus, the brain region responsible for memory formation, of the individual experiencing depression degenerates after prolonged exposure to elevated levels of corticosteroids [78,79].

Through focusing on WeChat use in a sample of older Chinese adults, the current study found that clear associations exist among social media use, depression, and memory performance among the elderly population, which has vital significance for theory and practice. On the one hand, this study extends the field of cognitive health by focusing on the association between social media use and memory performance among an age-specific group. Moreover, the findings of the data presented in this study provide new empirical support for the positive impact of social media use on the cognitive health of the elderly. In addition, by revealing the mediation effect of depression between social media use and memory performance, this study is helpful for furthering our understanding about how behavioral factors and lifestyle relate to memory change in the course of aging. On the other hand, through examining the association between social media use and memory performance and the operating mechanisms linking them, this study provides some useful insights into how the memories and cognitive health of elderly people could be improved. First, this study found that the use of social media may increase memory performance; therefore, it is necessary to promote social media among the elderly population and help them cross the digital divide. Accordingly, specific education programs aimed at helping the elderly to master the techniques and skills of using social media should be implemented. Second, alleviating the degree of depression was found to be the protective factor of memory performance. Therefore, we should study the psychological feelings of the elderly and adopt measures to make them happy and optimistic during the course of aging.

Although this study found that social media use had positive impacts on psychological health and cognitive health in older adults, the potential negative influences of social media use should not be ignored. In fact, some research has revealed that social media use, especially the problematic social media use, was significantly positively associated with certain health issues such as depression [44,45], anxiety [80,81], sleep disorders [82], and psychological distress [83,84]. Therefore, we should also pay attention to the possible negative consequences of using social media among older adults. Accordingly, education programs aimed at helping the elderly to avoid social media addiction and other problematic social media use should also be implemented.

This study analyzed a large and nationally representative sample including 4929 respondents, which guaranteed its external validity. However, this study also has some important limitations. First, this study used data obtained by a cross-sectional survey, which limited our ability to make causal statements among the variables of interest. Future research could adopt a longitudinal or experimental design to further explore the true cause-and-effect relationships. Second, the secondary data utilized in this study limited the selection of variables included in the research model. For example, many confounding factors, such as the time spent and activities undertaken on social media were not measured in the survey, so it was impossible to examine the relationship between memory performance and the specific social media engagement style among the elderly population. In the future, qualitative studies differentiating between various types of social media use could be adopted. Third, the measure of some variables did not adopt standardized forms, which limited the comparison with related studies. Fourth, social media use was measured as a dichotomy in this study, which neglected more important information related to the frequency of social media use. Future studies can adopt specialized rating scales to evaluate participants’ use of social media. Fifth, the evaluation of memory performance depended on a self-reported survey item, which was not sufficiently objective. As Crumley et al. pointed out, subjective memory belief measures cannot be a substitute for objective evaluations of memory [10]. In the future, multi-disciplinary efforts should be considered, and more rigorous and strict tests of memory ability should be adopted. Finally, memory decline in the course of aging involves changes in various types of memory; this study, however, failed to differentiate the types of memory due to using secondary data. Future studies can utilize experimental designs to explore the relationship between social media use and changes in specific types of memory. 

## 5. Conclusions

Changes to memory are influenced by various factors in the course of aging. Through focusing on WeChat use in a sample of older Chinese adults, the current study examined the association between social media use and memory performance and tested for the mediating role of depression. The results indicated that social media use positively correlated and depression negatively correlated with memory performance. Meanwhile, depression partially mediated the relationship between social media use and memory performance. This study found that using social media and alleviating depression may be the protective factor for memory performance, suggesting that social media and depression represent vital targets for interventions to delay the decline in memory in elderly people. In addition, through revealing the underlying mechanisms linking social media use to memory performance, this study provided a basis for future research on cognitive aging and cognitive health. 

## Figures and Tables

**Table 1:** Demographic characteristics of and WeChat usage rate in the sample.

Variables	N	Percentage (%)	WeChat Users (*n* = 993)	WeChat Usage Rate (%)	*p* (Chi-Squared Test)
Gender					
Male	2551	51.8	559	21.9	0.001
Female	2378	48.2	434	18.3	
Age group					
60–79	4694	95.2	974	20.7	<0.001
80+	235	4.8	19	8.1	
Marital status					
Never married	38	0.8	6	15.8	<0.001
Married (having a spouse)	4083	82.8	857	21.0	
Cohabitation	21	0.4	6	28.6	
Divorced	70	1.4	25	35.7	
Widowed	717	14.5	99	13.8	
Educational attainment					
Illiterate or semi-illiterate	1985	40.3	100	5.0	<0.001
Primary school	1110	22.5	136	12.3	
Junior high school	1056	21.4	340	32.2	
Senior high school (including vocational senior school)	638	12.9	322	50.5	
College or above	140	2.8	95	67.9	
Household registration type					
Agricultural	3342	67.8	358	10.7	<0.001
Non-agricultural	766	15.5	297	38.8	
Resident	810	16.4	336	41.5	
Others	11	0.2	2	18.2	

**Table 2:** Descriptive statistics for depression and memory performance scores by WeChat users and non-users.

Variables	Use of WeChat	N	Min	Max	Mean	SD	*t*	*p*
Depression	Users	993	8	32	12.44	3.96	−9.138	<0.001
	Non-users	3936	8	32	13.89	4.61		
Memory performance	Users	993	1	5	3.10	1.27	14.075	<0.001
	Non-users	3936	1	5	2.45	1.32		

Note: Min = minimal score, Max = maximal score, SD = standard deviation.

**Table 3:** Correlations among the variables of interest.

	WeChat Use	Depression	Memory Performance	Range	Mean	SD
WeChat use	1	−0.129 **	0.197 **	0, 1	0.20	0.40
Depression		1	−0.238 **	8–32	13.60	4.52
Memory performance			1	1–5	2.58	1.33

Note: SD = standard deviation, ** *p* < 0.01.

**Table 4:** Results of causal step regressions.

Regression	Path	Coefficient	Standardized Coefficient	SE	*p*	Constant	*t*
X–Y	c	0.373	0.279	0.052	<0.001	1.785	7.229
X–M	a	−0.833	−0.184	0.177	<0.001	16.560	−4.715
X–M–Y	b	−0.056	−0.190	0.004	<0.001	2.711	−13.708
	c′	0.326	0.244	0.051	<0.001	2.711	6.429

Note: X = WeChat use, M = depression, Y = memory performance, SE = standard error.

**Table 5:** Indirect, direct, and total effects and 95% confidence intervals (CI) for the mediation model.

No.	Effect	Value	95% CI	
			Lower	Upper
1	Indirect effect (WeChat use–depression–memory performance)	0.047	0.027	0.067
2	Direct effect (WeChat use–memory performance)	0.326	0.227	0.425
3	Total effect	0.373	0.272	0.474

## Data Availability

The data analyzed in this study are openly accessible on the website of the ISSS at Peking University (http://www.isss.pku.edu.cn/cfps/ (accessed on 22 May 2022)).
